# Supporting Parents During Cardiology Fellowship: Because Life Happens

**DOI:** 10.1161/JAHA.119.013392

**Published:** 2019-07-09

**Authors:** Jennifer L. Jarvie, Andrew E. Levy

**Affiliations:** ^1^ Division of Cardiology University of Colorado Anschutz Medical Campus Aurora CO

**Keywords:** Editorials, breastfeeding, Graduate Medical Education, fellowship, parental leave, pregnancy, training, women in cardiology, Pregnancy, Lifestyle, Women

## Abstract

See Article Mwakyanjala et al

In 1889, Johns Hopkins Hospital started the first medical residency program under the direction of Sir William Osler.^1^ Bright young medical school graduates—mostly men in their early 20s—were given a residence within the hospital for 1 to 3 years to complete their hands‐on training before practicing independently. Interns and residents received minimum compensation, lived in the hospital, and performed many “nonclinical chores” for the hospitals.^2^ The low wages and requirement to live in the hospital seemed reasonable for trainees, since it was assumed they had no outside obligations at such an early point in their lives and careers. However, by the mid‐20th century, medical education reform brought about the universal requirement for internships and residencies, a focus on education and competency testing, and a boom in required specialty training programs. Medical training expanded to include many specialties, sub‐specialties, and sub‐sub‐specialties—each with additional years of training. As the specialization of medicine increased, the average age of medical students increased from around 22 years of age at matriculation to 26 years in 2018.^3^ Women also began entering medical school in greater numbers, rising to represent almost 50% of medical school matriculates in 2018–2019.^3^ As expected, similar shifts are seen in postgraduate training, such that women represented 46% of all residents in 2018, up from just 19% in 1977.^4^ Board certifications, procedural requirements, competition, and an exponentially growing knowledge base have lengthened the average time spent in training, with most cardiovascular disease fellows spending 6 to 10 years in postgraduate training. The result is a modern generation of cardiovascular fellows who complete fellowship training in their mid‐30s, often while juggling personal life milestones such as marriage and parenthood.

In 2015, the Leadership Council for Women in Cardiology section of the American College of Cardiology sent out a decennial Professional Life Survey to investigate the demographics and barriers for professional satisfaction.^5^ A total of 2313 physicians (42% women, 58% men) responded to the survey. Compared with respondents to the 1996 survey, fewer cardiologists reported being single (14% women, 5% men) and more cardiologists reported having children (72% women, 87% men). While a majority of men continued to report having a spouse who provided childcare (57% men versus 13% women), women with children continued to rely on other caregivers for childcare. Overall trends, however, showed more women and men reported family responsibilities hindering their professional pursuits.^5^ That same year, the Women in Cardiology Pregnancy Workforce Work Group sent out a survey to American College of Cardiology women to determine how a career in cardiology impacts a woman's choices for family planning.^6^ A total of 501 women responded to the survey, with 76% reporting at least 1 pregnancy during their career. Half (49%) of women reported being pregnant during their cardiovascular fellowship. An additional 22% of women reported a pregnancy during residency and another 14% during their early career postfellowship. Taken together, this survey shows that a typical woman in cardiology will experience at least 1 pregnancy and become a parent during her training.

The current study by Mwakyanjala et al^7^ expands on this work in 2 important ways: first, by focusing on the experience of trainees and, second, by including male trainees in their sample. The study is limited by a small sample size (29 total trainees, 10 female trainees), but the information garnered is provocative. Nearly two thirds (59%) of cardiovascular trainees (men and women) reported having children, and all trainees with children reported having children during their training, extending the Women in Cardiology study findings to trainee fathers. Importantly, the study also found notable differences in the impact of cardiovascular training on female trainees compared with their male peers. Similar to findings by Poppas et al^8^ over 10 years ago, significantly more trainee mothers reported altering their training schedule to accommodate a pregnancy or maternity leave compared with their male counterparts (50% versus 20%, respectively). Women in the study also more frequently reported changing their schedule, in many cases, to avoid adverse exposures during a pregnancy (for example, avoiding radiation exposure in the cardiac catheterization lab or during nuclear cardiology rotations). Additionally, women in the study were more likely to report “front‐loading” their call before their expected delivery date, likely to minimize further sleep deprivation and allow for more consistent overnight breastfeeding and caregiving during early infancy.

More alarming is the finding that only half of cardiovascular trainee mothers continue breastfeeding beyond 6 months.^7^ This is in spite of a presumably better than average knowledge, and understanding, of the innumerable benefits of breastfeeding and the recommendation by the American Academy of Pediatrics that mothers breastfeed their infants exclusively for the first 6 months of life and continue for at least 1 year.^9^ One explanation for this pattern may relate to shorter maternity leave for trainee mothers, where prior investigators have found that maternity leave lasting <6 weeks contributes to shorter breastfeeding duration.^10^ Once mothers return to work, data from other specialties suggest that irregular pumping and feeding schedules at work, often with prolonged intervals between sessions, make it difficult or even impossible to maintain an adequate milk supply.^11^ By comparison, all but 1 (94%) of the women in the control group—composed of the nontrainee female spouses of trainees—breastfed beyond 6 months. Hospitals have made efforts to accommodate lactating mothers with on‐site lactation rooms; however, the implication is that new mothers in cardiovascular training are not given adequate time or opportunity to use them. More efforts are needed—by hospitals and training programs—to systematically address clinical demands and schedules to allow lactation breaks for physician mothers. Healthcare systems, training programs, and academic faculty who know well the benefits of breastfeeding should be the example to community employers and institutions for accommodating nursing mothers—not the exception.

Despite many parenting challenges during training, somewhat reassuringly, neither men nor women reported making significant changes in their career plans as a result of pregnancy or childbirth. Also, roughly equal numbers of mothers and fathers reported difficulty balancing parental responsibilities with research responsibilities and further study. A concerning finding from this survey was the near unanimous perception among both male and female cardiovascular trainee parents that their colleagues do not support them. More than two thirds of parents‐in‐training felt a stigma associated with pregnancy. Neither sex nor faculty‐versus‐trainee status seemed to influence this perception. However, both male and female trainee parents agreed that the perceived bias was worse for new mothers than new fathers.

These same respondents nearly unanimously reported inadequate parental leave policies, which provides insight into how these policies and program structure can contribute to perceived stigma. Under current rules established by the American Board of Internal Medicine, cardiovascular trainees are not to exceed more than “one month of time away from training, which includes vacation, illness, parental or family leave, or pregnancy‐related disabilities.”^12^ The result of this policy is that expectant trainee parents must stockpile their annual vacation time for parental leave and many trainees must also make arrangements for coverage during their absence (ideally with the help of the program), hoping to minimally inconvenience other trainees and avoid extending their own training. Often, parental‐leave coverage plans include pulling the remaining cardiovascular trainees off “easier” rotations to cover “essential”—typically more clinically demanding—rotations and call responsibilities on short notice because of a lack of forward planning by the program. To make matters worse, any inconveniences experienced by colleagues can never be fully mitigated since current strategies for “planning” parental leave include planning for childbirth on exact dates to fit with the timing of the trainee's vacation. This is obviously an inflexible and fallible system that does not allow for the natural variability surrounding pregnancy and delivery, and especially not for unexpected complications. Sarma et al^6^ found that almost half of cardiologist mothers experienced a pregnancy complication, so a system that fails to anticipate and provide plasticity for life events likely feeds perceived stigma.

A better system would recognize parental leave for trainees as separate and distinct from vacation.^13^ Paid parental leave policies are emerging at academic medical centers around the country, though few currently apply to trainees.^14^, ^15^ The treatment of trainees as “others” is troubling and creates a double standard that requires institutional recognition and reform. In the meantime, program directors alone are given the authority to adapt the American Board of Internal Medicine's policies, including the aforementioned “one‐month” policy, to fit the needs of individual trainees.^12^ In this light, program directors should feel empowered to draft parental leave policies in collaboration with trainees and faculty, so the expectations for coverage, time‐off, and emergency planning are explicit rather than implicit. In order to further minimize disruption to the program, and perceptions of inconvenience and stigma, the schedules of expectant trainees and coverage plans should be clearly outlined as early in the academic year as possible. Finally, cultivating a feeling of openness and even joy for expectant parents would not only combat stigma but also encourage and facilitate this early planning.

As a mentor once said, “Life continues to happen, even while you're in training.” Our profession needs to adapt to the changing demographics and needs of contemporary medical trainees while preserving the rigor and dedication required throughout medical training. Doing so is central to the continued success of our field. Historically, cardiology has struggled with the perception of being unsupportive of parents, especially mothers. That this perception deters talented individuals from pursuing cardiology because of a competing desire to grow a family harms the profession as a whole. Reversing this trend will be essential to attracting a new generation of medical students and residents, for whom flexibility at work and wellness in life increasingly constitute major considerations in their choice of specialty.^16^, ^17^ Perhaps we can start by acknowledging that most cardiovascular trainees will experience at least 1 pregnancy and become parents while in training. A pregnancy, therefore, should be something institutions and program directors anticipate and plan for; and while there will be growing pains associated with policies that support trainees’ time off for parental leave, in time this will lead to greater acceptance of parenthood during training, less disruption to training programs, and less perceived stigma against parents. More to the point: we should start to treat our trainees as professional colleagues and make allowances for their lives to grow and advance, just as their careers do. Because life continues to happen, even while you're in training.

## Sources of Funding

Dr Levy receives funding from National Institutes of Health (NIH) T32 Training Grant 5T32‐HL007822.

## Disclosures

None.
